# Supplementary material for: Skin Carotenoid Levels Are Associated with Demographic Factors, Body Size, and Fruit and Vegetable Intake in the Japanese Population
Source: Nutrients. 2024 Jul 4;16(13):2133. doi: 10.3390/nu16132133 (PMC11243080; doi:10.3390/nu16132133)
Supplement: Supplementary file 1 [file nutrients-16-02133-s001.zip › nutrients-3087797-supplementary.pdf]

Table S1. Categorisation of the number of vegetable dishes and the frequency of green, yellow, and light-coloured vegetable intake (n = 492)

		Number of vegetable dishes consumed (dish/day)												
		Almost never		1		2		3		4		≥5		
		n	(%)	n	(%)	n	(%)	n	(%)	n	(%)	n	(%)	*p*-Value
Frequency of green and yellow vegetable intake	≤ 1 time/week	18	(72.0)	21	(14.9)	5	(3.6)	1	(1.1)	1	(2.1)	1	(2.2)	<0.001
	2-3 time/week	4	(16.0)	50	(35.5)	26	(18.7)	9	(9.5)	2	(4.3)	0	(0.0)	
	4-6 time/week	3	(12.0)	44	(31.2)	50	(36.0)	22	(23.2)	8	(17.0)	4	(8.9)	
	≥ 1 time/day	0	(0.0)	26	(18.4)	58	(41.7)	63	(66.3)	36	(76.6)	40	(88.9)	
Frequency of light-coloured vegetable intake	≤ 1 time/week	15	(60.0)	17	(12.1)	4	(2.9)	2	(2.1)	1	(2.1)	1	(2.2)	<0.001
	2-3 time/week	5	(20.0)	47	(33.3)	17	(12.2)	10	(10.5)	1	(2.1)	1	(2.2)	
	4-6 time/week	5	(20.0)	54	(38.3)	52	(37.4)	11	(11.6)	6	(12.8)	1	(2.2)	
	≥ 1 time/day	0	(0.0)	23	(16.3)	66	(47.5)	72	(75.8)	39	(83.0)	42	(93.3)	

A chi-square test was used.

Table S2. Categorisation of the number of vegetable dishes consumed and their corresponding food items (n = 492)

		Number of vegetable dishes consumed (dish/day)												
		Almost never		1		2		3		4		≥5		
		n	(%)	n	(%)	n	(%)	n	(%)	n	(%)	n	(%)	*p*-Value
Frequency of food group intake in the last month														
Rice	≤ 2-3	3	(12.0)	10	(7.1)	7	(5.0)	3	(3.2)	2	(4.3)	2	(4.4)	0.209
	4-6 times/week	7	(28.0)	21	(15.0)	20	(14.4)	9	(9.5)	4	(8.5)	4	(8.9)	
	≥ 1 time/day	15	(60.0)	109	(77.9)	112	(80.6)	83	(87.4)	41	(87.2)	39	(86.7)	
Breads	≤ 1 time/week	13	(52.0)	43	(30.7)	43	(31.6)	31	(33.3)	12	(26.1)	15	(36.6)	0.893
	2-3 times/week	6	(24.0)	39	(27.9)	36	(26.5)	24	(25.8)	12	(26.1)	7	(17.1)	
	4-6 times/week	2	(8.0)	19	(13.6)	21	(15.4)	13	(14.0)	6	(13.0)	6	(14.6)	
	≥ 1 time/day	4	(16.0)	39	(27.9)	36	(26.5)	25	(26.9)	16	(34.8)	13	(31.7)	
Noodles	< 1 time/week	6	(24.0)	34	(24.8)	32	(24.1)	21	(22.6)	8	(17.4)	15	(35.7)	0.121
	1 time/week	5	(20.0)	41	(29.9)	43	(32.3)	28	(30.1)	22	(47.8)	16	(38.1)	
	2-3 times/week	9	(36.0)	37	(27.0)	43	(32.3)	33	(35.5)	9	(19.6)	4	(9.5)	
	≥ 4-6	5	(20.0)	25	(18.3)	15	(11.3)	11	(11.8)	7	(15.2)	7	(16.7)	
Meats	≤ 1 time/week	5	(20.0)	7	(5.0)	9	(6.5)	3	(3.2)	1	(2.2)	2	(4.4)	0.001
	2-3 times/week	7	(28.0)	45	(31.9)	40	(29.0)	17	(18.1)	4	(8.9)	5	(11.1)	
	4-6 times/week	8	(32.0)	50	(35.5)	50	(36.2)	33	(35.1)	19	(42.2)	17	(37.8)	
	≥ 1 time/day	5	(20.0)	39	(27.7)	39	(28.3)	41	(43.6)	21	(46.7)	21	(46.7)	
Fish and shellfish	≤ 1 time/week	11	(44.0)	52	(37.1)	30	(21.7)	13	(13.7)	6	(13.0)	3	(6.7)	<0.001
	2-3 times/week	12	(48.0)	55	(39.3)	64	(46.4)	43	(45.3)	14	(30.4)	10	(22.2)	
	4-6 times/week	1	(4.0)	25	(17.9)	28	(20.3)	20	(21.1)	15	(32.6)	15	(33.3)	
	≥ 1 time/day	1	(4.0)	8	(5.7)	16	(11.6)	19	(20.0)	11	(23.9)	17	(37.8)	
Eggs	≤ 1 time/week	8	(32.0)	28	(19.9)	22	(15.8)	7	(7.5)	4	(8.7)	6	(13.3)	0.194
	2-3 times/week	8	(32.0)	50	(35.5)	43	(30.9)	33	(35.1)	14	(30.4)	12	(26.7)	
	4-6 times/week	4	(16.0)	33	(23.4)	37	(26.6)	22	(23.4)	14	(30.4)	14	(31.1)	
	≥ 1 time/day	5	(20.0)	30	(21.3)	37	(26.6)	32	(34.0)	14	(30.4)	13	(28.9)	

Soybeans and soybean products	≤ 1 time/week	15	(60.0)	39	(27.9)	25	(18.3)	10	(10.6)	2	(4.3)	5	(11.1)	<0.001
	2-3 times/week	6	(24.0)	43	(30.7)	37	(27.0)	23	(24.5)	13	(27.7)	6	(13.3)	
	4-6 times/week	2	(8.0)	33	(23.6)	32	(23.4)	24	(25.5)	10	(21.3)	12	(26.7)	
	≥ 1 time/day	2	(8.0)	25	(17.9)	43	(31.4)	37	(39.4)	22	(46.8)	22	(48.9)	
Fruits	≤ 1 time/week	17	(68.0)	68	(48.2)	46	(33.1)	25	(26.3)	12	(25.5)	8	(17.8)	<0.001
	2-3 times/week	5	(20.0)	38	(27.0)	32	(23.0)	22	(23.2)	5	(10.6)	6	(13.3)	
	4-6 times/week	1	(4.0)	13	(9.2)	32	(23.0)	13	(13.7)	11	(23.4)	8	(17.8)	
	≥ 1 time/day	2	(8.0)	22	(15.6)	29	(20.9)	35	(36.8)	19	(40.4)	23	(51.1)	
Milk and dairy products	≤ 1 time/week	10	(40.0)	45	(31.9)	23	(16.6)	21	(22.1)	6	(13.0)	3	(6.7)	<0.001
	2-3 times/week	3	(12.0)	33	(23.4)	24	(17.3)	11	(11.6)	3	(6.5)	0	(0.0)	
	4-6 times/week	5	(20.0)	23	(16.3)	26	(18.7)	12	(12.6)	8	(17.4)	8	(17.8)	
	≥ 1 time/day	7	(28.0)	40	(28.4)	66	(47.5)	51	(53.7)	29	(63.0)	34	(75.6)	

A chi-square test was used.

Table S3. Categorisation of the frequency of fruit intake and their corresponding food items (n = 492)

		Frequency of fruits intake								
		≤ 1 time/week		2-3 times/week		4-6 times/week		≥ 1 time/day		
		n	(%)	n	(%)	n	(%)	n	(%)	p-Value
Frequency of food group intake in the last month										
Rice	≤ 2-3 times/week	17	(9.7)	4	(3.7)	0	(0.0)	6	(4.7)	<0.001
	4-6 times/week	37	(21.0)	13	(12.0)	9	(11.5)	6	(4.7)	
	≥ 1 time/day	122	(69.3)	91	(84.3)	69	(88.5)	117	(90.7)	
Breads	≤ 1 times/week	66	(38.2)	26	(24.1)	24	(32.0)	41	(32.8)	<0.001
	2-3 times/week	50	(28.9)	40	(37.0)	15	(20.0)	19	(15.2)	
	4-6 times/week	23	(13.3)	20	(18.5)	15	(20.0)	9	(7.2)	
	≥ 1 time/day	34	(19.7)	22	(20.4)	21	(28.0)	56	(44.8)	
Noodles	< 1 time/week	40	(23.5)	19	(17.8)	16	(20.8)	41	(33.6)	0.067
	1 time/week	55	(32.4)	44	(41.1)	26	(33.8)	30	(24.6)	
	2-3 times/week	49	(28.8)	34	(31.8)	23	(29.9)	29	(23.8)	
	≥ 4-6 times/week	26	(15.3)	10	(9.4)	12	(15.6)	22	(18.0)	
Meats	≤ 1 time/week	11	(6.3)	4	(3.7)	4	(5.2)	8	(6.2)	0.014
	2-3 times/week	45	(25.9)	27	(25.0)	17	(22.1)	29	(22.5)	
	4-6 times/week	72	(41.4)	42	(38.9)	33	(42.9)	30	(23.3)	
	≥ 1 time/day	46	(26.4)	35	(32.4)	23	(29.9)	62	(48.1)	
Fish and shellfish	≤ 1 time/week	74	(42.5)	20	(18.5)	12	(15.6)	9	(6.9)	<0.001
	2-3 times/week	69	(39.7)	54	(50.0)	33	(42.9)	42	(32.3)	
	4-6 times/week	23	(13.2)	28	(25.9)	21	(27.3)	32	(24.6)	
	≥ 1 time/day	8	(4.6)	6	(5.6)	11	(14.3)	47	(36.2)	
Eggs	≤ 1 time/week	43	(24.6)	15	(13.9)	9	(11.7)	8	(6.2)	<0.001
	2-3 times/week	71	(40.6)	26	(24.1)	21	(27.3)	42	(32.3)	
	4-6 times/week	36	(20.6)	40	(37.0)	26	(33.8)	22	(16.9)	
	≥ 1 time/day	25	(14.3)	27	(25.0)	21	(27.3)	58	(44.6)	

Soybeans and soybean products	≤ 1 time/week	59	(33.9)	18	(16.7)	11	(14.1)	8	(6.3)	<0.001
	2-3 times/week	54	(31.0)	36	(33.3)	19	(24.4)	19	(14.8)	
	4-6 times/week	35	(20.1)	31	(28.7)	24	(30.8)	23	(18.0)	
	≥ 1 time/day	26	(14.9)	23	(21.3)	24	(30.8)	78	(60.9)	
Green and yellow vegetables	≤ 1 time/week	34	(19.3)	6	(5.6)	4	(5.1)	3	(2.3)	<0.001
	2-3 times/week	57	(32.4)	19	(17.6)	8	(10.3)	7	(5.4)	
	4-6 times/week	45	(25.6)	38	(35.2)	30	(38.5)	18	(13.9)	
	≥ 1 time/day	40	(22.7)	45	(41.7)	36	(46.2)	102	(78.5)	
Light-coloured vegetables	≤ 1 time/week	33	(18.8)	2	(1.9)	2	(2.6)	3	(2.3)	<0.001
	2-3 times/week	46	(26.1)	22	(20.4)	5	(6.4)	8	(6.2)	
	4-6 times/week	47	(26.7)	39	(36.1)	28	(35.9)	15	(11.5)	
	≥ 1 time/day	50	(28.4)	45	(41.7)	43	(55.1)	104	(80.0)	
Milk and dairy products	≤ 1 time/week	67	(38.3)	21	(19.4)	10	(12.8)	10	(7.7)	<0.001
	2-3 times/week	26	(14.9)	32	(29.6)	7	(9.0)	9	(6.9)	
	4-6 times/week	28	(16.0)	17	(15.7)	25	(32.1)	12	(9.2)	
	≥ 1 time/day	54	(30.9)	38	(35.2)	36	(46.2)	99	(76.2)	

A chi-square test was used.
